# Clinical criteria accurately diagnose severe but not moderate alcohol-associated hepatitis: A systematic review and meta-analysis

**DOI:** 10.1097/HC9.0000000000000404

**Published:** 2024-03-18

**Authors:** Nipun Verma, Rohit Mehtani, Jacob Martin Haiar, Pranita Pradhan, Ajay Duseja, Gene Young Im, Ashwani K. Singal

**Affiliations:** 1 Department of Hepatology, Postgraduate Institute of Medical Education and Research, Chandigarh, India; 2 Department of Hepatology, Amrita Institute of Medical Sciences and Research, Faridabad, Haryana, India; 3 Department of Internal Medicine, University of California, San Diego, California, USA; 4 Department of Pediatrics, Indian Council of Medical Research Center for Evidence-Based Child Health, Postgraduate Institute of Medical Education and Research, Chandigarh, India; 5 Department of Medicine, Division of Liver Diseases, Recanati/Miller Transplantation Institute, The Icahn School of Medicine at Mount Sinai, New York, New York, USA; 6 Department of Medicine, Division of Gastroenterology and Hepatology, University of Louisville School of Medicine, Louisville, Kentucky, USA; 7 Department of Medicine, Jewish Hospital and Trager Transplant Center, Louisville, Kentucky, USA; 8 Department of Medicine, VA Medical Center Sioux Falls, South Dakota, USA

## Abstract

**Background:**

The precision of clinical criteria and the utility of liver biopsy for diagnosis or prognosis remain unclear in patients with alcohol-associated hepatitis (AH). We systematically reviewed the literature to answer these questions.

**Methods:**

Four databases were searched for studies describing the precision of clinical criteria (National Institute on Alcohol Abuse and Alcoholism, European Association for Study of Liver, or classical) and the role of histology in AH. The precision(positive predictive value) of criteria was pooled through random-effects meta-analysis, and its variation was investigated through subgroups and meta-regression of study-level factors with their percent contribution to variation (*R* ^2^). The risk of bias among studies was evaluated through the QUADAS2 tool (*PROSPERO-ID-CRD4203457250*).

**Results:**

Of 4320 studies, 18 in the systematic review and 15 (10/5: low/high risk of bias, N=1639) were included in the meta-analysis. The pooled precision of clinical criteria was 80.2% (95% CI: 69.7–89.7, *I* ^2^:93%, *p* < 0.01), higher in studies with severe AH (mean-Model for End-Stage Liver Disease > 20) versus moderate AH (mean-Model for End-Stage Liver Disease < 20): 92% versus 67.1%, *p* < 0.01, and in studies with serum bilirubin cutoff 5 versus 3 mg/dL (88.5% vs.78.8%, *p* = 0.01). The factors contributing to variation in precision were Model for End-Stage Liver Disease (*R* ^2^:72.7%), upper gastrointestinal bleed (*R* ^2^:56.3%), aspartate aminotransferase:aspartate aminotransferase ratio (*R* ^2^:100%), clinical criteria (*R* ^2^:40.9%), bilirubin (*R* ^2^:22.5%), and Mallory body on histology (*R* ^2^:19.1%).

The net inter-pathologist agreement for histologic findings of AH was variable (0.33–0.97), best among 2 studies describing AH through simple and uniform criteria, including steatosis, ballooning, and neutrophilic inflammation. Few studies reported the utility of histology in estimating steroid responsiveness (N = 1) and patient prognosis (N = 4); however, very broad septa, pericellular fibrosis, and cholestasis were associated with mortality. Bilirubinostasis was associated with infection in 1 study.

**Conclusions:**

Clinical criteria are reasonably precise for diagnosing severe AH, while there is an unmet need for better criteria for diagnosing moderate AH. Histologic diagnosis of AH should be simple and uniform.

## Graphical abstract

**Figure:**
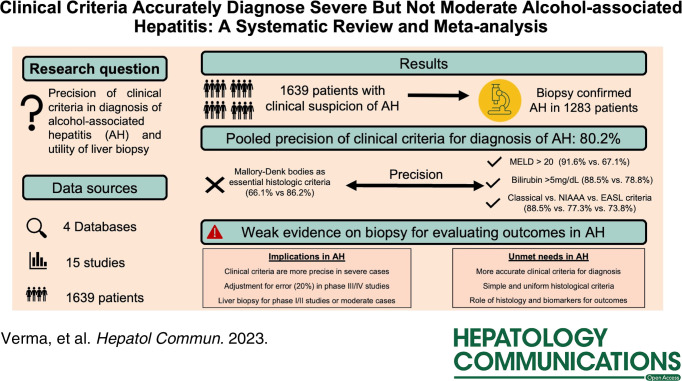

## INTRODUCTION

Alcohol-associated hepatitis (AH) is the most severe form of alcohol-associated liver disease, which is associated with high short-term mortality.^1^,^2^ Until few years ago, the criteria for the clinical diagnosis of AH were variable. Often, it was defined as a recent onset of jaundice with/without other features of decompensation in a patient with ongoing alcohol abuse and/or features of steatohepatitis on liver biopsy.^3^,^4^ Further, due to the invasiveness and limited availability of liver biopsy in routine clinical practice, the diagnosis was often based on clinical and laboratory parameters alone, with liver biopsy reserved when clinical diagnosis was uncertain.^3^,^4^

Since the criteria for diagnosis of AH were heterogeneous, the National Institute on Alcohol Abuse and Alcoholism (NIAAA) in 2016 developed clinical criteria for AH based on expert consensus.^5^ These criteria are the development of jaundice within the prior 8 weeks with serum bilirubin > 3 mg/dL, ongoing alcohol consumption (> 40 grams/day for females and > 60 grams/day for males) for ≥ 6 months within at least prior 60 days from onset of jaundice, aspartate aminotransferase (AST) to alanine aminotransferase ratio > 1.5 with both values < 400 IU/L and AST > 50 IU/L.^5^. Based on these criteria,^5^ a patient can be stratified to have (a) definite AH when all clinical criteria are met, and the liver biopsy is consistent with AH, (b) probable AH when all clinical criteria are met without available liver histology, and (c) possible AH when one or more clinical criteria are not met due to presence of confounding factors. Based on these criteria, patients with definite and probable AH were considered candidates for steroid therapy and entry into clinical trials.^5^ Despite widespread use, very few studies have investigated the diagnostic role of clinical criteria for AH. Variability in diagnostic accuracy of clinical criteria and interobserver variability in the reporting of liver biopsy findings add to the heterogeneity in the clinical trial enrollment and in the clinical diagnosis of AH in practice.

Therefore, we conducted this systematic review and meta-analysis to ascertain the precision of clinical criteria for diagnosing AH on liver histology. We also explored the utility of liver biopsy in risk stratification and predicting infection and response to steroids in patients with AH.

## METHODS

### Study design

We performed the study according to the PRISMA guidelines,^6^ and the protocol was registered in PROSPERO (*CRD4203457250*).

### Outcomes of study

The primary objective was to evaluate the precision or positive predictive value (outcome) of clinical criteria of AH (index test) with liver biopsy (as reference standard) in patients (> 18 y) who consume alcohol in significant amounts per day (population). The precision in each study was calculated as the proportion of patients with clinical criteria diagnosed to have AH on histology. The other objectives of the study were to (a) evaluate the utility of liver biopsy in estimating prognosis, infection, and response to steroids and (b) examine inter-pathologist reliability for diagnosing AH.

### Literature search

Three independent investigators (Nipun Verma, Rohit Mehtani, Jacob Martin Haiar) selected studies for the analysis from the list of articles retrieved through an electronic search conducted by a librarian (Pranita Pradhan) in PubMed, EBSCO, Scopus, and Embase from their inception until November 30, 2023. The search strategy used Boolean combinations of the clinical and MeSH terms, including alcohol consumers, liver biopsy, diagnosis, and prognosis, as illustrated in the Supplemental data, http://links.lww.com/HC9/A833.

### Eligibility criteria

Observational or interventional studies reporting patients (>18 years) with a history of significant alcohol consumption, with clinical suspicion of AH, and availability of liver biopsy results were included in the study, and the availability of liver biopsy results. Duplicate articles, studies with insufficient data (conference proceedings, meeting abstracts, and case reports), and non-English language were excluded. Grey literature search and the references in original articles and published reviews were manually screened for additional studies. Studies reporting histological confirmation of AH in clinically suspected patients were included in the meta-analysis.

### Data extraction

Three investigators (Nipun Verma, Rohit Mehtani, and Jacob Martin Haiar) independently extracted the data utilizing a pre-piloted data extraction sheet. The disagreements in study selection and data extraction were resolved through discussion with the arbitrator (Ashwani K. Singal). Clinical suspicion of AH was mainly based on rapid deterioration in liver functions, including jaundice and elevated aminotransferases in a patient with heavy alcohol use (Tables 1–3). Briefly, these clinical criteria were itemized into the European Association for Study of Liver 2012 criteria,^7^ Classical criteria,^8^ and the NIAAA criteria^5^ (Table 1).

**TABLE 1:** Clinical criteria for diagnosis of alcohol-associated hepatitis

References	Clinical presentation	Serum bilirubin (mg/dL)	Other laboratory data	Alcohol use
NIAAA criteria^5^	Jaundice	> 3	AST/ALT > 1.5 and < 400 IU/L	> 40 g/d in women and > 60 g/d in men within previous at least 8 wk
EASL criteria^7^	Recent jaundice±ascites	Not defined	Not defined	Ongoing
Classical criteria^8^	Ascites	> 5	AST/ALT > 2 and > 300 IU/L, neutrophilia, elevated INR	Heavy

Abbreviations: ALT, alanine aminotransferase; AST, aspartate aminotransferase; EASL, European Association for Study of Liver; INR, international normalized ratio; NIAAA, National Institute of Alcoholism and Alcohol Abuse.

**TABLE 2:** Characteristics of included studies

References, country	Design	Criteria AH^a^	Inclusion criteria for biopsy	Excluded patients	Biopsy criteria for AH	Cirrhosis on biopsy (%)	Route, timing, and interpreters of biopsy	Overall ROB
Mookerjee^12^ UK	Prospective	EASL 2012	Acute decompensation of ALD (ascites, edema, and jaundice), cirrhosis on clinical and radiology, alcohol use > 80 g/d (men), > 60 g/d (women)	< 18 or > 75 y, other etiology of liver disease, severe organ dysfunction, malignancy, and infection	Hepatocellular ballooning, ASH grading	75.0	Within 7 d of admission, primarily by transjugular route, by 2 pathologists, blinded	Low
Hardy^13^ UK	Prospective	EASL 2012	Recent jaundice with excessive alcohol intake, DF ≥ 32	Infection, SBP, UGI Bleed, Cardio-respiratory disease	Not clear	81.0	Route, timing not clear, 1 expert pathologist	High
Altamirano^9^ UK and USA	Prospective	EASL 2012	Alcohol > 60 g/day, moderately elevated bilirubin, AST > ALT, elevated GGT	HCC, any other liver disease	Hepatocellular ballooning, Mallory bodies, inflammatory infiltrate (predominantly polymorphonuclear), and pericellular fibrosis	82.0	Within 48 hours of admission by transjugular route, 1 liver pathologist was blinded	Low
Rudler^15^ France	Prospective	EASL 2012	Jaundice, Bilirubin> 2.9 mg/dl, excessive alcohol consumer, DF ≥ 32	Other etiology of acute or chronic liver disease	Steatosis, ballooning, and neutrophil infiltration	100.0	Within 3 days of admission, the route was not clear, and a single pathologist blinded	High
Rudler^14^ France	Retrospective	EASL 2012	Jaundice, bilirubin > 2.9 mg/dl, < 3 mo, DF ≥32, active drinking, long term alcoholism	Advanced HCC, other etiology of cirrhosis, HIV, and severe comorbidities	Neutrophil infiltrate and 1: ballooning or Mallory bodies	100.0	Timing not clear, transjugular route, rest not clear	High
Roth^16^ USA	Retrospective	Classical criteria	Bilirubin > 5 mg/dl, chronic alcoholism, jaundice < 3 mo, and DF ≥ 32	Insufficient biopsy, duration > 14 d after admission, > 7 d after steroids, prior biopsy, HIV, nonalcoholic, nonhepatic causes of liver disease	Ballooning and lobular inflammation±Mallory bodies	61.0	Median 6 d (range: 1–14) after admission by transjugular (85%) or percutaneous (15%) routes. Liver pathologist (1), blinded	Low
Shetty^17^ India	Retrospective	NIAAA	Jaundice, 18–65 y age, alcohol intake > 50 g/day, > 6 mo, < 2 mo abstinence, DF > 32	Other causes of liver disease viz. Infection, viral hepatitis, malignancy, AST/ALT > 400 U/l	Steatosis, hepatocyte ballooning, Mallory hyaline bodies, and neutrophilic inflammation	33.0	Within 7 d of admission by transjugular route, 1 pathologist	High
Bissonnette^18^ France, Spain	Prospective	EASL 2012	Alcohol consumer > 60 g/day, moderately elevated AST, ALT, GGT, AST > ALT, elevated bilirubin	HCC, active extrahepatic malignancy, prior liver transplant, prior TIPS	Hepatocellular ballooning and Mallory-Denk bodies, inflammatory infiltrate (predominantly polymorphonuclear cells), and pericellular fibrosis	NA	Within 48 h of admission, transjugular route, 3 liver histopathologists	Low
Shasthry^19^ India	Prospective	Classical criteria	Bil > 5 mg/dl, heavy alcohol consumer, within 60 days before jaundice, AST/ALT elevation, < 500 IU/mL, AST: ALT ≥ 2, INR1.5, neutrophilia	Infections, sepsis, UGIB, renal insufficiency, tuberculosis, acute viral hepatitis, DILI, nonconsenting patients	Ballooning and lobular inflammation	100.0	Time not clear, transjugular, by 2 liver pathologists	Low
Choudhary^20^ India	Retrospective	NIAAA	Bil > 3 mg/dl, probable AH as per NIAAA posted for transplant	Other causes of liver disease.	Steatosis, one or more ballooning degeneration, Mallory hyaline bodies, neutrophilic infiltration, and pericellular fibrosis	95.0	Not clear	High
Lee^21^ Korea	Prospective	Unclear	≥ 18 y of age, alcohol > 60 g/day for men and > 40 g/day for women, elevated GGT, and AST, ALT, AST: ALT > 1	Other etiologies of chronic liver disease	Mallory bodies, hepatocellular ballooning), inflammatory infiltrate polymorphonuclear leukocytes, and pericellular fibrosis	76.0	Within 7 d of admission, percutaneous or transjugular route	Low
Dubois^22^ Switzerland, Belgium	Prospective	NIAAA	Bil > 2.9 mg/dl, alcohol consumer > 60 g/day (men), > 40 g/day (women), < 90 d jaundice, DF > 32, < 60 d of abstinence, and histologic AH	Viral hepatitis, AIH, hemochromatosis, Co-infection with hepatitis A, B, C, or E	Steatosis, ballooning, and neutrophilic infiltration	93.0	Time, route not clear, by 2 liver pathologists	Low
Atkinson^23^ UK	Retrospective	Classical criteria	Bilirubin > 4.7 mg/dL, alcohol use (> 80 g/d for males, > 60 g/d for females), presentation in < 4 wk, longstanding alcohol misuse, clinical, laboratory, DF ≥ 32	No other identified causes for their liver disease, abstinence > 2 mo, AST > 500, ALT > 300 IU/L	Hepatocellular ballooning, Mallory bodies, inflammatory infiltrate (predominantly polymorphonuclear cells), and pericellular fibrosis		Timing not clear, transjugular route, by 2 blinded histopathologists	Low
Forrest^24^ UK	Retrospective	Classical criteria	Bil > 4.7 mg/dl, alcohol > 80 g/day(men), > 60 g/day (women), < 4 wk, DF ≥ 32	Abstinence > 2 mo, jaundice > 3 mo, other liver diseases, malignancies, steroid or pentoxifylline use in last 6 weeks, AST > 500, ALT > 300, creatinine > 500mmol/L, RRT, inotropes, GI bleed, untreated sepsis, cerebral hemorrhage, extensive retinal hemorrhage, acute myocardial infarction, arrhythmia, pregnancy, and lactating women	Hepatocyte steatosis, ballooning, and inflammatory infiltration	71.0	Within 48 hours of admission, transjugular route, 2 blinded histopathologists.	Low
Avitabile^25^ Spain, USA	Prospective	NIAAA	Bil > 3 mg/dl, probable AH as per NIAAA	< 18 or > 85 y, previous liver or kidney transplantation, HCC, other liver diseases, sepsis, insufficient liver biopsy, and lack of consent	Steatosis (any degree), hepatocellular ballooning, and lobular inflammation	69.0	The timing and route are not clear. Two blinded liver pathologists	Low
Horvath^26^ USA	Retrospective	NA	Biopsy-proven AH	Not clear	Not clear	NA	Time, route not clear, by 5 blinded pathologists	NA
Andrade^27^ Portugal	Retrospective	Unclear	> 18 y, alcohol consumer > 80 g/day (men), > 60 g/day (women), AST, ALT: 5–10× ULN, AST > ALT, elevated GGT, bilirubin	Other causes of liver disease	Not clear	NA	Within 7 days of admission, transjugular, 2 liver pathologists	NA
Lackner^28^ Multicenter Europe	Retrospective	NA	Biopsy-proven ALD, compensated, decompensated, and histologic ASH	NA	SALVE grading: Ballooning and neutrophil scores >= 1 each with activity score >= 2	NA	Time and route are not clear, at least 3 pathologists	NA
						**Pooled:** 85.1% (71.6–92.9), *I* ^2^ 85%, *p* < 0.01		

a Clinical criteria: EASL 20127: “Recent onset of jaundice and/or ascites in a patient with ongoing alcohol misuse”, NIAAA 20165: “Jaundice (> 3 mg/dl), within last 8 weeks, with AST/ALT >1.5, both < 400IU/L, in patients with ongoing alcohol use > 40 g/d(females), > 60 g/day(males), excluding other acute and chronic liver diseases”, Classical criteria8 (Lucey, NEJM 2009): “Abnormal LFT with bilirubin > 5 mg/dL, AST/ALT > 2; both < 300IU/L, elevated INR, neutrophilia in patients with ascites and heavy alcohol abuse.”

Abbreviations: AH, alcohol-associated hepatitis; AIH, autoimmune hepatitis; ALD, alcohol-associated liver disease; ALT, alanine aminotransferase; ASH, alcohol-associated steatohepatitis; AST, aspartate aminotransferase; Bil, bilirubin; DF, discriminant function; GGT, gamma-glutamyl transferase; LFT, liver function test; NA, not available; NIAAA, National Institute on Alcohol Abuse and Alcoholism; ROB, risk of bias; SBP, pontaneous bacterial peritonitis; RRT, renal replacement therapy; UGI, upper gastrointestinal; ULN, upper limit normal.

**TABLE 3:** Pooled precision of clinical criteria for diagnosis of alcohol-associated hepatitis on histology in various study-level subgroups

Attributes	Subgroup (No. studies)	Cases with clinical diagnosis	Precision	95% CI	Heterogeneity (*I* ^*2*^)	Sub-group difference (*p*-value)
Study characteristics	Origin of study					0.98
	West (11 studies)^9^,^12^,^13^,^14^,^15^,^16^,^18^,^22^,^23^,^24^,^25^	1314	80.2	70.1–87.5	91	
	East (4 studies)^17^,^19^,^20^,^21^	325	79.9	43.2–95.4	96	
	Design					0.65
	Prospective (9 studies)^9^,^12^,^13^,^15^,^18^,^19^,^21^,^22^,^25^	955	82.5	66.5–91.8	91	
	Retrospective (6 studies)^14^,^16^,^17^,^20^,^23^,^24^	684	78.0	60.9–89.0	95	
	Random sampling					0.68
	Yes	882	78.3	62.9–88.6	91	
	No	757	82.2	65.4–91.9	94	
Population	MDF above 32					0.14
	Yes (8 studies)^13^,^15^,^16^,^17^,^22^,^23^,^24^	871	85.1	78.1–90.1	76	
	No or unclear (7 studies)^9^,^12^,^18^,^19^,^20^,^21^,^25^	768	72.3	50.6–86.9	93	
	MELD above 20^a^					<0.01
	Yes (3 studies)^15^,^22^,^23^	317	91.6	87.3–94.6	56	
	No (4 studies)^12^,^16^,^18^,^25^	490	67.1	46.6–82.7	93	
	Leucocytosis above 11000/ul					0.97
	Yes (5 studies)^12^,^13^,^16^,^17^,^19^	401	78.9	54.3–92.2	92	
	No (6 studies)^9^,^14^,^18^,^21^,^23^,^25^	746	78.5	67.5–86.5	88	
Index test	Clinical criteria^b^					0.04
	EASL (6 studies)^9^,^12^,^13^,^14^,^15^,^18^	688	73.8	59.2–84.5	91	
	NIAAA (4 studies)^17^,^20^,^22^,^25^	337	77.3	29.3–96.6	90	
	Classical criteria (4 studies)^16^,^19^,^23^,^24^	493	88.5	85.2–91.1	47	
	Unclear (1 study)^21^	121	88.4	81.3–93.5		
	Bilirubin levels					0.01
	> 3 mg/dl or >50 μmol/L (6 studies)^14^,^15^,^17^,^20^,^22^,^25^	593	78.8	52.6–92.6	94	
	> 5 mg/dl or 80 μmol/L (4 studies)^16^,^19^,^23^,^24^	493	88.5	85.2–91.1	47	
	Unclear (5 studies)^9^,^12^,^13^,^18^,^21^	553	71.6	55.1–83.8	91	
Reference test	Biopsy criteria					
	Ballooning and inflammation (8 studies)^9^,^12^,^13^,^16^,^18^,^19^,^21^,^23^	885	81.3	67.6–90.1	92	0.87
	Steatosis, ballooning, and inflammation (7 studies)^14^,^15^,^17^,^20^,^22^,^24^,^25^	754	79.6	59.3–91.3	94	
	Mallory-Denk bodies					0.02
	Essential (5 studies)^9^,^14^,^17^,^18^,^20^	570	66.0	50.2–78.9	92	
	No or unclear (10 studies)^12^,^13^,^15^,^16^,^19^,^21^,^22^,^23^,^24^,^25^	1069	86.1	74.8–92.8	91	
	Pericellular fibrosis					0.45
	Essential (5 studies)^9^,^18^,^20^,^21^,^23^	615	75.1	52.9–89.0	95	
	No or unclear (10 studies)^12^,^13^,^14^,^15^,^16^,^17^,^19^,^22^,^24^,^25^	1024	82.9	70.0–90.9	90	
	Reporting by 2 or more pathologists					0.50
	Yes (7 studies)^12^,^18^,^19^,^22^,^23^,^24^,^25^	746	84.9	62.7–95.0	93	
	No or unclear (8 studies)^9^,^13^,^14^,^15^,^16^,^17^,^20^,^21^	893	78.0	65.5–86.8	93	
	Reporting by the liver pathologist					0.34
	Yes (8 studies)^9^,^13^,^16^,^18^,^19^,^21^,^22^,^25^	936	84.2	70.9–92.2	89	
	No or unclear (7 studies)^12^,^14^,^15^,^17^,^20^,^23^,^24^	703	75.3	56.5–87.7	95	
Quality of study	Risk of bias					0.26
	Low (10 studies)^9^,^12^,^16^,^18^,^19^,^21^,^22^,^23^,^24^,^25^	1194	83.9	71.1–91.7	92	
	High (5 studies)^13^,^14^,^15^,^17^,^20^	445	72.6	51.9–86.7	94	

a Data on MELD score of patients meeting clinical criteria for AH available in 7 studies,

b Clinical criteria: EASL 2012:^7^ “Recent onset of jaundice and/or ascites in a patient with ongoing alcohol misuse,” NIAAA 2016:^5^ “Jaundice (>3 mg/dl), within last 8weeks, with AST/ALT > 1.5, both < 400 IU/L, in patients with ongoing alcohol use > 40 g/d(females), > 60 g/day(males), excluding other acute and chronic liver diseases”, Classical criteria^8^ (Lucey, NEJM 2009): “Abnormal LFT with bilirubin > 5 mg/dL, AST/ALT > 2; both < 300IU/L, elevated INR, neutrophilia in patients with ascites and heavy alcohol abuse”.

Abbreviations: AH, alcohol-associated hepatitis; ALT, alanine aminotransferase; AST, aspartate aminotransferase; EASL, European Association for Study of Liver; INR, international normalized ratio; LFT, liver function test; MDF, Maddrey’s discriminant function; MELD, Model for End-stage Liver Disease; NIAAA, National Institute on Alcohol Abuse and Alcoholism.

For each study, the following variables were extracted: (a) study details: author’s name, country, publication year, design-prospective or retrospective; (b) demographics: age and gender; (c) clinical data: cases with clinical suspicion of AH, criteria of suspicion, excluded patients, ascites, HE, and upper gastrointestinal bleed; (d) histology data: liver biopsy findings, criteria for histological diagnosis, cases with histologically confirmed AH, route, timing and interpreter(s) of liver biopsy; d) laboratory data: leukocyte count, Maddrey discriminant function, Model for End-Stage Liver Disease (MELD), Child Turcotte Pugh, serum bilirubin, international normalized ratio, serum creatinine, serum AST and alanine aminotransferase, and serum albumin. Interobserver reliability (kappa coefficient) between pathologists for diagnosing AH, neutrophilic infiltration, bilirubinostasis, fibrosis stage, and megamitochondria was noted.

Alcohol-associated hepatitis histologic score (AHHS)^9^ (Supplemental Table S1, http://links.lww.com/HC9/A833) and its utility for mortality risk assessment, infection prediction, and steroid response were recorded. As one study used the Consortium for the Study of Alcohol-related LiVer Disease in Europe (SALVE) criteria for grading (Supplemental Table S2, http://links.lww.com/HC9/A833) and staging (Supplemental Table S3, http://links.lww.com/HC9/A833), these criteria were noted with their implications for the diagnosis and prognosis of AH. Any discrepancy during data collection was resolved through discussion with an arbitrator (Ashwani K. Singal). Finally, 2 investigators (Rohit Mehtani and Nipun Verma) independently assessed the study quality using the QUADAS2 tool^10^ and resolution of conflicts with an arbitrator (Ashwani K. Singal). This tool evaluated the risk of bias (ROB) in 4 domains, viz., patient selection, index test, reference standard, and patient flow. To be eligible for a low ROB, a study had to score low ROB in 3 or more domains.

### Statistical analysis

The mean (±SD) or number (%) were described as appropriate. A generalized linear mixed model^11^ was conducted to pool the estimates of diagnostic precision for each study, where estimates were logit-transformed, pooled, and retransformed to proportions during random effect meta-analysis. Restricted maximum likelihood method with Biggerstaff and Jackson method for confidence intervals and Hartung-Knapp adjustment were used for estimating between study variances. The heterogeneity in estimates was assessed by Tau^2^, *I* ^2^, and chi-square test (Q-statistic). The cutoffs for small, medium, and large heterogeneity (*I* ^2^) were > 25%, 50%, and 75%, respectively. A list of study-level attributes specific to design, population, index test, reference standard, and quality of study was used to investigate heterogeneity in estimates within subgroups and on meta-regression using a mixed-effects model. Outlier study assessments were performed to identify studies contributing to heterogeneity. Leave-one study-out plots were created for sensitivity analysis. The funnel plot and Egger’s regression were done to assess the asymmetry in estimates. All analysis was performed using the R studio v. 1.4.1103, and a *p*-value of < 0.1 was considered significant for heterogeneity and < 0.05 for subgroups and meta-regression.

## RESULTS

**FIGURE 1:**
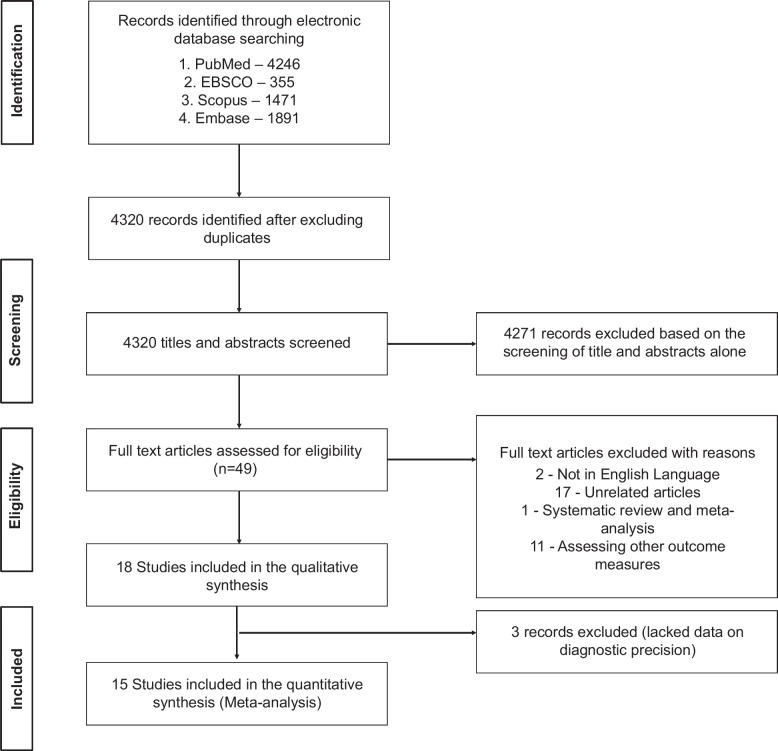
PRISMA flow chart.

Of the 7963 references identified, 4320 were screened after excluding duplicate records, and 49 full texts were assessed for eligibility after excluding articles based on title and abstracts. Thirty-one articles were excluded due to various reasons (Figure 1). Finally, 18 studies^9^,^12^,^13^,^14^,^15^,^16^,^17^,^18^,^19^,^20^,^21^,^22^,^23^,^24^,^25^,^26^,^27^,^28^ were included in the systematic review. Three studies^26^,^27^,^28^ did not provide data on the precision of clinical criteria for AH, leaving 15 studies for the quantitative meta-analysis.

### Study characteristics

Of 18 studies, 9 were retrospective,^14^,^16^,^17^,^20^,^23^,^24^,^26^,^27^,^28^ and 9 were prospective studies.^9^,^12^,^13^,^15^,^18^,^19^,^21^,^22^,^25^ Of 15 studies included in the meta-analysis, 4 studies used NIAAA criteria,^17^,^20^,^22^,^25^ 4 used Classical criteria,^16^,^19^,^23^,^24^ 6 used European Association for Study of Liver 2012 criteria,^9^,^12^,^13^,^14^,^15^,^18^ while 1 study did not report as to which criteria were used.^21^ Eight studies^13^,^14^,^15^,^16^,^17^,^22^,^23^,^24^ included patients with severe AH as defined by a discriminant function (DF ≥ 32). Criteria used to define AH on histology were also heterogeneous. Steatosis, hepatocyte ballooning, and inflammation (predominantly neutrophilic) were used to diagnose AH in 7 studies,^14^,^15^,^17^,^20^,^22^,^24^,^25^ while 8 studies^9^,^12^,^13^,^16^,^18^,^19^,^21^,^23^ did not include steatosis as an essential criterion for diagnosis of AH. Timing of liver biopsy after admission was within 48 hours in 3 studies,^9^,^18^,^24^ within 3 days in 1 study,^15^ within 7 days in 4 studies,^12^,^17^,^21^,^27^ and not defined in 9 studies.^13^,^14^,^16^,^19^,^20^,^22^,^23^,^25^,^26^ A single pathologist evaluated liver biopsy in 4 studies,^9^,^15^,^16^,^17^ 2 pathologists in 6 studies,^12^,^19^,^22^,^23^,^25^,^27^ more than 2 pathologists in 2 studies^18^,^26^ while this was not defined in 5 studies.^13^,^14^,^20^,^21^,^24^ The study characteristics are summarized in Table 2 and Supplemental Table S4, http://links.lww.com/HC9/A833.

### Outcomes

#### Precision of clinical criteria for diagnosis of AH

**FIGURE 2:**
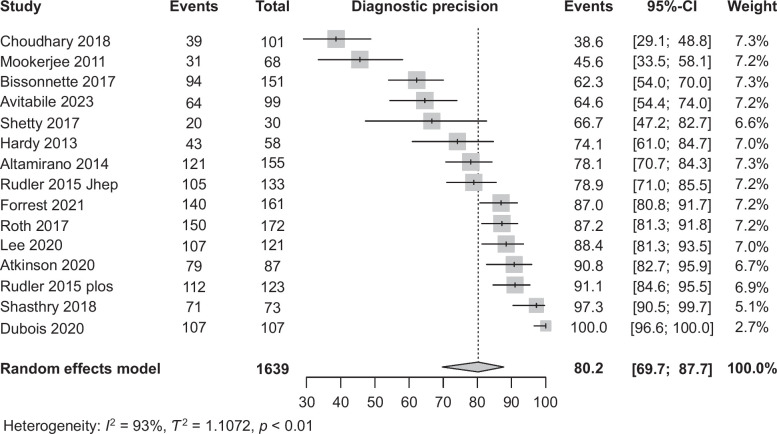
Forest plot showing pooled precision of clinical criteria for diagnosis of alcohol-associated hepatitis.

Of 1639 patients from 15 studies,^9^,^12^,^13^,^14^,^15^,^16^,^17^,^18^,^19^,^20^,^21^,^22^,^23^,^24^,^25^ with clinical suspicion of AH, biopsy confirmation was documented in 1283 patients. The pooled precision was 80.2% (95% CI: 69.7–89.7, Figure 2). However, there was high heterogeneity between studies (*p* < 0.01; *I* ^2^ = 93%).

#### Heterogeneity evaluation

The pooled precision of clinical criteria was higher among studies with mean-MELD scores > 20 than those with lower MELD scores (91.6% vs. 67.1%, *p* < 0.01) (Table 3). Precision was also numerically higher in studies including only patients with Maddrey discriminant function scores > 32 (85.1% vs. 72.3%), although this was not significant, *p* = 0.14. Based on the clinical criteria, the precision was 88.5% for Classical criteria (88.5%), 77.3% for NIAAA criteria, and 73.8% for European Association for Study of Liver criteria, *p* = 0.04. Serum bilirubin > 5 mg/dL (80 μmol/L) versus 3 mg/dL (50 μmol/L) was also associated with improved precision of clinical diagnosis of AH (88.5% vs. 78.8%, *p* = 0.01). Further, including Mallory bodies in the histologic criteria of AH was associated with a lower precision of clinical diagnosis of AH (66.1% vs. 86.1%, *p* = 0.02). There were no differences in precision estimates based on prospective or retrospective study design, random or nonrandom patient recruitment, region, leukocytosis, biopsy criteria, reporting by 1 or 2 pathologists, or assessment by an expert liver pathologist (Table 3).

Investigating further the variation in precision of clinical criteria, we delved into relevant study-level factors (identified a-priori and through subgroup analysis) to assess their contribution to heterogeneity, both in terms of quantity (*R* ^2^) and direction (beta coefficients) through meta-regression. Our findings revealed that MELD (contribution: 72.7%), upper gastrointestinal bleed (contribution: 56.3%), AST to ALT ratio (contribution: 100%), clinical criteria (contribution: 40.9%), bilirubin (contribution: 22.5%), and Mallory bodies on histology (contribution: 19.1%) emerged as significant contributors to the variations in pooled estimates(Table 4).

**TABLE 4:** Meta-regression to investigate heterogeneity in the pooled estimate of precision of clinical criteria for diagnosing alcohol-associated hepatitis

Variable in study	*R* ^2^ (% contribution to heterogeneity)	Beta coefficient	95% CI	*p* ^a^
MELD (mean)	72.7	0.261	0.104 to 0.418	0.001
MDF>32	16.1	0.901	−0.145 to 1.947	0.091
UGI bleed (proportion)	56.3	0.056	0.010 to 0.101	0.018
AST: ALT ratio (mean)^b^	100.0	4.940	3.374 to 6.506	<0.001
Clinical criteria	40.9			0.034
EASL^7^		Ref.		
NIAAA^5^		−0.334	−1.509 to 0.842	0.578
Classical criteria^8^		1.263	0.123 to 2.402	0.029
Baseline bilirubin	22.5			
> 3 mg/dl or > 50 μmol/l		Ref.		
> 5 mg/dl or 80 μmol/l		1.159	−0.126 to 2.443	0.077
Unclear		−0.227	−1.400 to 0.946	0.704
Mallory bodies as an essential criterion	19.1	−1.121	−2.191 to -0.050	0.040

*Notes*: *R* ^2^ quantifies the extent to which study-level factors contribute to heterogeneity in pooled estimates. Beta coefficient conveys both the strength and direction of the impact on the pooled estimates of the precision of clinical criteria for each unit change for a numerical variable or factor comparison to a reference category for categorical factors. For example, an increase of 1 U in the MELD score is associated with an increase in precision estimates by ~0.261 U, while the presence of classical criteria in comparison to EASL criteria would increase the precision estimates by 1.263 U. The presence of Mallory bodies is associated with a decrease in precision estimates by ~1.121 U.

Other variables that were nonsignificant (*p* > 0.05) were CTP, MDF, age, WBC, bilirubin, INR, creatinine, albumin, ascites, HE, and male gender.

a Mixed effect model with restricted maximum likelihood estimator. #10 studies with available data.

b Data from studies15,18,19.

Abbreviations: ALT, alanine aminotransferase; AST, aspartate aminotransferase; CTP, Child Turcotte Pugh score; EASL, European Association for Study of Liver; INR, international normalized ratio; MDF, Maddrey’s discriminant function; MELD, Model for End-Stage Liver Disease; NIAAA, National Institute on Alcohol Abuse and Alcoholism; UGI, upper gastrointestinal; WBC, white blood cell count.

Outlier analysis did not reveal any influential study (Supplemental Figure 1, http://links.lww.com/HC9/A833). Leave-one analysis showed the precision of clinical criteria after removing each study from the meta-analysis (Supplemental Figure 2, http://links.lww.com/HC9/A833).

#### Role of histology in risk stratification

Seven studies evaluated the role of liver biopsy in predicting patient prognosis (Table 5).^9^,^15^,^16^,^21^,^22^,^27^,^28^ Of these, 3 studies^9^,^21^,^27^,^28^ reported higher mortality in patients with high AHHS scores. One study reported cholestasis, cirrhosis with very broad septa, and severe pericellular fibrosis as predictors of poor survival.^28^ Three studies^9^,^22^,^27^ analyzed the response to steroids based on the AHHS score. One study^27^ demonstrated a better response in patients with low AHHS scores, while the other 2 studies^9^,^22^ did not show any difference. Modified AHHS predicted higher mortality in patients with MELD < 21 and age, serum bilirubin, INR, and serum creatinine - (ABIC) score class A and B.^9^,^21^ Bilirubinostasis predicted risk infection risk in 1 study^9^ (OR 1.57; 95% CI: 1.00–2.47; *p* = 0.04).

**TABLE 5:** Role of histology in risk stratification of patients with alcohol-associated hepatitis

References	Mortality risk stratification	Added value to clinical scores	Infection prediction	Response to steroids
Altamirano^9^	Ninety-day survival: mild (17/17, 100%) vs. moderate AHHS (20/24, 83%) (*p* = 0.08) and moderate vs severe AHHS (44/68, 65%) (*p* = 0.007).	Patients with ABIC class B stratified by modified AHHS^b^ into 2 risk groups for survival at 90 days: low risk (36/38, 95%) vs. high risk (94/135, 70%) (*p* = 0.003). In patients with MELD < 21, also stratified into 2 risk groups: low risk (52/55, 94%) vs. high risk (84/116, 72%) (*p* = 0.001)	Bilirubinostasis (OR 1.57, 1.00–2.47, *p* = 0.04).	No difference in AHHS between lille responders and nonresponders (5.2 [2.0) vs. 6.0 [2.0], *p*=0.20)
Rudler^14^	Two-year survival is not different between moderate (42%) and severe AHHS (38%) (*p* = 0.90).	NA	NA	NA
Andrade^27^	AHHS is associated with 90-day mortality (OR 2.78, 1.37–5.34, *p* < 0.001). AHHS > 7 100% sensitivity, 100% specificity, AUC 1.00, *p* < 0.001	NA	NA	Steroid responders vs. nonresponders: AHHS was lower (5.4 [0.9] vs. 8.1[1.1], *p*=0.003). AHHS < 6: steroid responders with AUC 0.900, 0.742–1.000; sensitivity 87.5%, specificity 90%, PPV 98%, NPV 90%, (*p* = 0.004)
Roth^16^	Not associated with 60-day (HR: 1.01, 0.82–1.26, *p*=0.86) or 180-day mortality (HR: 1.03, 0.84–1.25, *p* = 0.79)	NA	NA	NA
Dubois^22^	Not associated with survival. Survival in the mild, moderate, and severe AHHS groups were 90%, 72%, and 69% at 28 days (*p*=0.6), 80%, 52%, and 63% at 3 mo (*p*=0.3), and 70%, 41% and 58% at 6 mo (*p*=0.3), respectively	NA	NA	Did not predict steroid response. The response was 50%, 73%, and 58% in the mild, moderate, and severe AHHS groups, respectively (*p* = 0.3)
Lee^21^	Traditional AHHS score^a^ is not associated with survival (moderate vs mild: HR: 1.57, 0.67–3.66, *p* = 0.300), (severe vs. mild: HR: 2.37, 1.00–5.59, *p* = 0.050), and (severe vs. moderate: HR: 1.51, 0.83–2.73, *p* = 0.176). Modified score^b^: High vs. low risk (HR: 3.16, 1.65–6.07, *p* = 0.001).	Modified AHHS^b^ did not improve the mortality prediction in patients with a MELD score ≥ 21 (*p* = 0.262). However, it discriminated risk of death in patients with ABIC class A and B (ABIC score < 9) (*p* = 0.001) and patients with MELD score < 21 (*p* = 0.006)	NA	NA
Lackner^28^	Canalicular and ductular cholestasis was associated with poor 90-day survival (*p* = 0.029). Cirrhosis with very broad septa (SALVE stage 4C) were associated with poor 10-year (*p* < 0.001) and 90-day survival (*p* = 0.070) Severe pericellular fibrosis associated with poor survival, liver injury, inflammation, fibro-obliterative venous lesions, and cholestasis	Cirrhosis with very broad septa (SALVE stage 4C) was independent predictor of 90-day mortality (adjusted HR: 2.16, 1.11–4.18, *p* = 0.023) in addition to MELD and HE	NA	NA

a Tradition score refers to AHHS mild (0–3), moderate (4–5), and severe (6–9).

b Modified score refers to AHHS low risk (0–4) and high risk (5–9).

Abbreviations: ABIC, age, serum bilirubin, INR and serum creatinine; AHHS, alcohol-associated hepatitis histologic score; MELD, Model for End-Stage Liver Disease; NPV, negative predictive value; PPV, positive predictive value.

#### Biopsy findings in patients without AH

Five studies^13^,^16^,^17^,^18^,^24^ reported biopsy findings in patients not confirmed to have AH on liver biopsy. The majority of these patients had alcohol-induced cirrhosis on liver biopsy. Other findings included cholestasis, hypoxic hepatitis, alcoholic foamy degeneration, steatosis with advanced fibrosis, and infectious mononucleosis hepatitis (Supplemental Table S5, http://links.lww.com/HC9/A833).

#### Interobserver agreement between pathologists

Four studies^9^,^16^,^22^,^26^ reported the interobserver agreement between pathologists who assessed AHHS. The net interobserver agreement ranged from 0.33 to 0.97 (Table 6). The kappa coefficient was lowest for megamitochondria (up to 0.2)^26^ and highest for fibrosis staging (up to 1.0).^22^ SALVE criteria used in 1 study^28^ showed an interobserver coefficient of 0.8 for fibrosis, 0.67 for neutrophilic infiltration, and 0.65–0.66 for canalicular or ductular cholestasis. Two studies,^22^,^25^ with simple histological criteria of steatosis (ballooning, and inflammation with neutrophils) had the best interobserver agreement (Table 6).

**TABLE 6:** Interobserver agreement between pathologists assessing histology in alcohol-associated hepatitis

References	Pathologists	Megamitochondria	Fibrosis stage	PMN infiltrate	Bilirubinostasis	Net value
Altamirano^9^	One expert liver pathologist, slides cross shared between central pathologist	0.46 (0.27–0.65)	0.65 (0.36–0.94)	0.6 (0.42–0.78)	0.86 (0.75–0.97)	0.64 (0.53–0.80)
Horvath^26^	Three expert liver pathologists and 2 luminal GI pathologists	0.2 (0.03–0.46)	0.42 (0.31–0.51)	0.52 (0.40–0.68)	0.52 (0.36–0.72)	0.33 (0.20–0.51)
Roth^16^	Three expert liver pathologists			0.65 (0.55–0.73)		
Dubois^22^	Two expert liver pathologists	0.46	1	0.9	0.74	0.67
Avitabile^25^	Two expert liver pathologists					0.97
Lackner^28^	More than 3 pathologists		0.80	0.67	0.33^a^, 0.65^b^, 0.66^c^	

*Notes*: Numerical values represent the kappa coefficient (95% CI).

a Hepatocellular cholestasis.

b Canalicular cholestasis.

c Ductular cholestasis.

Abbreviation: GI, gastrointestinal; PMN, polymorphonuclear cells.

#### Quality assessments

The ROB was high in 5 studies^13^,^14^,^15^,^17^,^20^ and low in the remaining 10 studies^9^,^12^,^16^,^18^,^19^,^21^,^22^,^23^,^24^,^25^ included in the quantitative meta-analysis (Supplemental Table S6, http://links.lww.com/HC9/A833). There was no difference in precision estimates of clinical criteria between studies with high and low ROB (Table 3).

#### Publication bias

Funnel plot (Supplemental Figure 3, http://links.lww.com/HC9/A833) and Egger’s regression with SE as a predictor of log-odds of precision showed asymmetry (*z* = 3.6036, *p* = 0.0003). Further, using the study’s sample size as a predictor instead of SE abolished the asymmetry (*z* = 0.660, *p* = 0.509), suggesting a small study effect as a plausible reason for asymmetry.

## DISCUSSION

### Main findings

This is the first meta-analysis that identified the precision of clinical criteria for diagnosing AH. Interestingly, studies including patients with severe disease (MELD > 20) or serum bilirubin cutoff of 5 mg/dL had a higher precision than those with a lower MELD score or serum bilirubin cutoff of 3 mg/dL. Further, the inclusion of Mallory bodies as a histology criterion for AH reduced the precision estimate of the clinical diagnosis of AH.

### Variations in the precision of clinical criteria for diagnosing AH and its implications

Although clinical criteria seem reasonably accurate in diagnosing AH in 92% of individuals with severe disease (MELD > 20), about a third of patients with moderate AH (MELD < 20) may not have AH on histology and would merit a liver biopsy to confirm AH. Given a significant 90 d mortality risk of up to 10%–20% in patients with moderate AH^2^, investigators recruit these patients in clinical trials for drug development for AH. Our study has an impact on the sample size of studies recruiting patients with moderate AH based on the clinical criteria, as there would be a need to adjust the sample size by a margin of 33%. Although previous studies^16^,^25^ showed improved precision of clinical criteria through the addition of inflammatory markers. For example, in 1 study^25^ performance of NIAAA criteria showed a sensitivity of 63%, specificity of 78%, and negative predictive values of 77% in AH diagnosis, and the accuracy of NIAAA criteria improved by adding c-reactive protein values. However, we did not find any difference in precision estimates based on leukocytosis. This could be due to heterogeneity of data or selection bias, as most of the included patients may have received corticosteroids, affecting the patient’s leukocyte counts. Further, given imprecision rates of about 20%–30% for clinical criteria and to avoid false discoveries, we believe patients recruited for phase I/II trials should be confirmed through biopsy before enrolment. While for the phase III/IV trials, one can adjust for error rates of 20% in sample size estimations for robust analysis.

### Liver biopsy for diagnosing AH

Liver biopsy, although considered the gold standard for AH diagnosis, has its limitations including but not limited to bleeding, pain, and physician or patient reluctance. Biopsy findings considered specific for AH may also be seen in other conditions, such as ballooning and necrosis in ischemia, Mallory-Denk bodies in cholestasis, and polymorphonuclear infiltrate in sepsis.^29^ Further, variations in defining AH on histology and on interobserver agreement also limit its applicability. For example, in the current study, having Mallory bodies as a mandatory criterion for histologic diagnosis of AH reduced the precision of clinical criteria in diagnosing AH. Reporting by an expert liver pathologist or >1 pathologist did not impact the precision of AH diagnosis. In fact, the greater number of pathologists involved in reporting unclear histological criteria led to the poorest interobserver reliability (0.33).^26^ Two studies using simple histologic criteria (steatosis, ballooning of hepatocytes, and lobular inflammation with neutrophils) had the best interobserver agreement between pathologists,^22^,^25^ suggesting a need for simple and uniform criteria for defining AH on histology. The SALVE group^28^ also used uniform histological criteria for AH diagnosis and showed good reliability. However, the SALVE criteria are not validated in other studies.

### Liver biopsy for risk stratification in AH

The role of liver biopsy for risk stratification in AH is unclear. In the current analysis, only 2 studies^9^,^27^ observed a higher short-term mortality in patients with higher AHHS scores. It has been earlier shown that AHHS may be better in the risk stratification in patients with moderate AH with MELD < 21 and/or ABIC < 9.^9^,^21^ Very broad septa and severe pericellular fibrosis were linked to mortality in patients with AH.^28^ Cirrhosis with very broad septa was an independent predictor of mortality with MELD and HE in patients with AH.^28^ Bilirubinostasis type (hepatocellular, ductal, or canalicular) and fibrosis stage were independent predictors of a poor prognosis.^28^ Bilirubinostasis, a surrogate marker for impaired hepatocellular bile transport and hepatic bile flow in AH, has also been associated with the development of bacterial infection and sepsis,^9^ which may contribute to increased mortality.

### Strengths and limitations

Although our study provides novel insights into the diagnostic precision of clinical criteria and the role of liver biopsy in patients with AH, we do recognize certain limitations of our study. We showed that studies with mean-MELD > 20 had better precision; however, this was primarily derived from high bilirubin rather than international normalized ratio or creatinine levels that may independently contribute to higher MELD scores. Therefore, we believe MELD should not be used as defining criteria for AH. Heterogeneity in estimates, nonuniform diagnostic criteria, retrospective study design across studies, small study effects, and publication bias could have affected the results. Lack of information on receipt of corticosteroids before the liver biopsy in studies limited analysis of the corticosteroid treatment on the precision of clinical criteria on AH diagnosis.^24^,^30^ We could not perform multi-variable meta-regression to ascertain the interaction of multiple factors as a reason for variation in estimates due to the limited number of studies. Finally, due to limited studies, the evidence on histology for estimating prognosis, steroid response, and infection prediction in AH remains weak and dictates the need for further evidence.

## CONCLUSIONS

The clinical criteria are precise in diagnosing AH in 80% of cases with better precision (92%) among severe cases with bilirubin > 5 mg/dL and/or MELD > 20, suggesting that AH is not only a histologic diagnosis. There remains an unmet need for better criteria for diagnosing AH in patients with moderate severity. Further studies are needed to (a) derive more accurate clinical criteria for diagnosing AH (b) define AH on histology through simple and uniform criteria, and (c) establish the role of histology in predicting outcomes in patients with AH. Finally, given the risks associated with liver biopsy, noninvasive tests and biomarkers are urgently needed to improve diagnosis and risk stratification in patients with AH.

## Supplementary Material
