# Supplementary material for: Clinical criteria accurately diagnose severe but not moderate alcohol-associated hepatitis: A systematic review and meta-analysis
Source: Hepatol Commun. 2024 Mar 18;8(4):e0404. doi: 10.1097/HC9.0000000000000404 (PMC10948131; doi:10.1097/HC9.0000000000000404)
Supplement: Supplementary file 1 [file hc9-8-e0404-s001.docx]

**Supplementary data**

**Precision of clinical criteria and role of liver biopsy for the diagnosis and prognosis of alcohol-associated hepatitis: a systematic review and meta-analysis**

Nipun Verma^1*^, Rohit Mehtani^2^, Jacob Martin Haiar^3^, Pranita Pradhan^4^, Ajay Duseja^1^, Gene Young Im^5^, Ashwani Singal^6,9*^

*^1^Department of Hepatology, Postgraduate Institute of Medical Education and Research, Chandigarh, India, ^2^Department of Hepatology, Amrita Institute of Medical Sciences and Research, Faridabad, Haryana, India, ^3^Department of Internal Medicine, University of California, San Diego, California, USA, ^4^Department of Pediatrics, Indian Council of Medical Research Center for Evidence Based Child Health, Postgraduate Institute of Medical Education and Research, Chandigarh, India, ^5^Recanati/Miller Transplantation Institute, Division of Liver Diseases, Department of Medicine, The Icahn School of Medicine at Mount Sinai, New York, New York. ^6^Department of Medicine, Division of Gastroenterology and Hepatology, University of Louisville School of Medicine, Louisville, Kentucky, USA; ^9^Jewish Hospital and Trager Transplant Center.*

*Joint corresponding authors

**Corresponding authors**

Ashwani K. Singal MD, MS, FACG, FAASLD, AGAF

Professor of Medicine, University of Louisville School of Medicine

R-505, 505 S Hancock St. Louisville, KY 40202

502-587-4358 (O) 502-587-4879 (Fax), 502-852-2902 (Research)

Email: [ashwani.singal@louisville.edu](mailto:ashwani.singal@louisville.edu), [ashwanisingal.com@gmail.com](mailto:ashwanisingal.com@gmail.com)

And

Dr. Nipun Verma, MD, DM, DipML

Associate Professor, Department of Hepatology

Postgraduate Institute of Medical Education and Research, Sector 12, Chandigarh, India, 160012

Email: [nipun29j@gmail.com](mailto:nipun29j@gmail.com), [verma.nipun@pgimer.edu.in](mailto:verma.nipun@pgimer.edu.in), Phone: 0172-2754777

**Search strategy**

**PUBMED**

| Search | Actions | Details | Query | Results | Time |
| --- | --- | --- | --- | --- | --- |
| #14 |  |  | Search: (((("Hepatitis, Alcoholic"[Mesh]) OR (Alcoholic hepatitis)) OR ("alcohol associated hepatitis")) OR ("alcohol related hepatitis")) AND ((((histology) OR (biopsy)) OR (histopathology)) OR ("liver biopsy pathology")) | [4,102](https://pubmed.ncbi.nlm.nih.gov/?term=%28%28%28%28%22Hepatitis%2C+Alcoholic%22%5BMesh%5D%29+OR+%28Alcoholic+hepatitis%29%29+OR+%28%22alcohol+associated+hepatitis%22%29%29+OR+%28%22alcohol+related+hepatitis%22%29%29+AND+%28%28%28%28histology%29+OR+%28biopsy%29%29+OR+%28histopathology%29%29+OR+%28%22liver+biopsy+pathology%22%29%29&sort=relevance&size=200) | 00:25:54 |
| #13 |  |  | Search: (((histology) OR (biopsy)) OR (histopathology)) OR ("liver biopsy pathology") | [6,441,975](https://pubmed.ncbi.nlm.nih.gov/?term=%28%28%28histology%29+OR+%28biopsy%29%29+OR+%28histopathology%29%29+OR+%28%22liver+biopsy+pathology%22%29&sort=relevance&size=200) | 00:25:35 |
| #12 |  |  | Search: "liver biopsy pathology" | [18](https://pubmed.ncbi.nlm.nih.gov/?term=%22liver+biopsy+pathology%22&sort=relevance&size=200) | 00:24:48 |
| #8 |  |  | Search: histopathology | [4,012,780](https://pubmed.ncbi.nlm.nih.gov/?term=histopathology&sort=relevance&size=200) | 00:23:37 |
| #7 |  |  | Search: biopsy | [4,204,102](https://pubmed.ncbi.nlm.nih.gov/?term=biopsy+&sort=relevance&size=200) | 00:23:21 |
| #6 |  |  | Search: histology | [5,529,886](https://pubmed.ncbi.nlm.nih.gov/?term=histology+&sort=relevance&size=200) | 00:22:57 |
| #5 |  |  | Search: ((("Hepatitis, Alcoholic"[Mesh]) OR (Alcoholic hepatitis)) OR ("alcohol associated hepatitis")) OR ("alcohol related hepatitis") | [10,312](https://pubmed.ncbi.nlm.nih.gov/?term=%28%28%28%22Hepatitis%2C+Alcoholic%22%5BMesh%5D%29+OR+%28Alcoholic+hepatitis%29%29+OR+%28%22alcohol+associated+hepatitis%22%29%29+OR+%28%22alcohol+related+hepatitis%22%29&sort=relevance&size=200) | 00:22:28 |
| #4 |  |  | Search: "alcohol related hepatitis" | [39](https://pubmed.ncbi.nlm.nih.gov/?term=%22alcohol+related+hepatitis%22&sort=relevance&size=200) | 00:22:10 |
| #3 |  |  | Search: "alcohol associated hepatitis" | [105](https://pubmed.ncbi.nlm.nih.gov/?term=%22alcohol+associated+hepatitis%22&size=200&sort=relevance) | 00:21:37 |
| #2 |  |  | Search: Alcoholic hepatitis | [10,274](https://pubmed.ncbi.nlm.nih.gov/?term=Alcoholic+hepatitis+&sort=relevance&size=200) | 00:20:40 |
| #1 |  |  | Search: "Hepatitis, Alcoholic"[Mesh] Sort by: Most Recent | [2,521](https://pubmed.ncbi.nlm.nih.gov/?sort=date&term=%22Hepatitis%2C+Alcoholic%22%5BMesh%5D&size=200) | 00:20:07 |

**EBSCO**


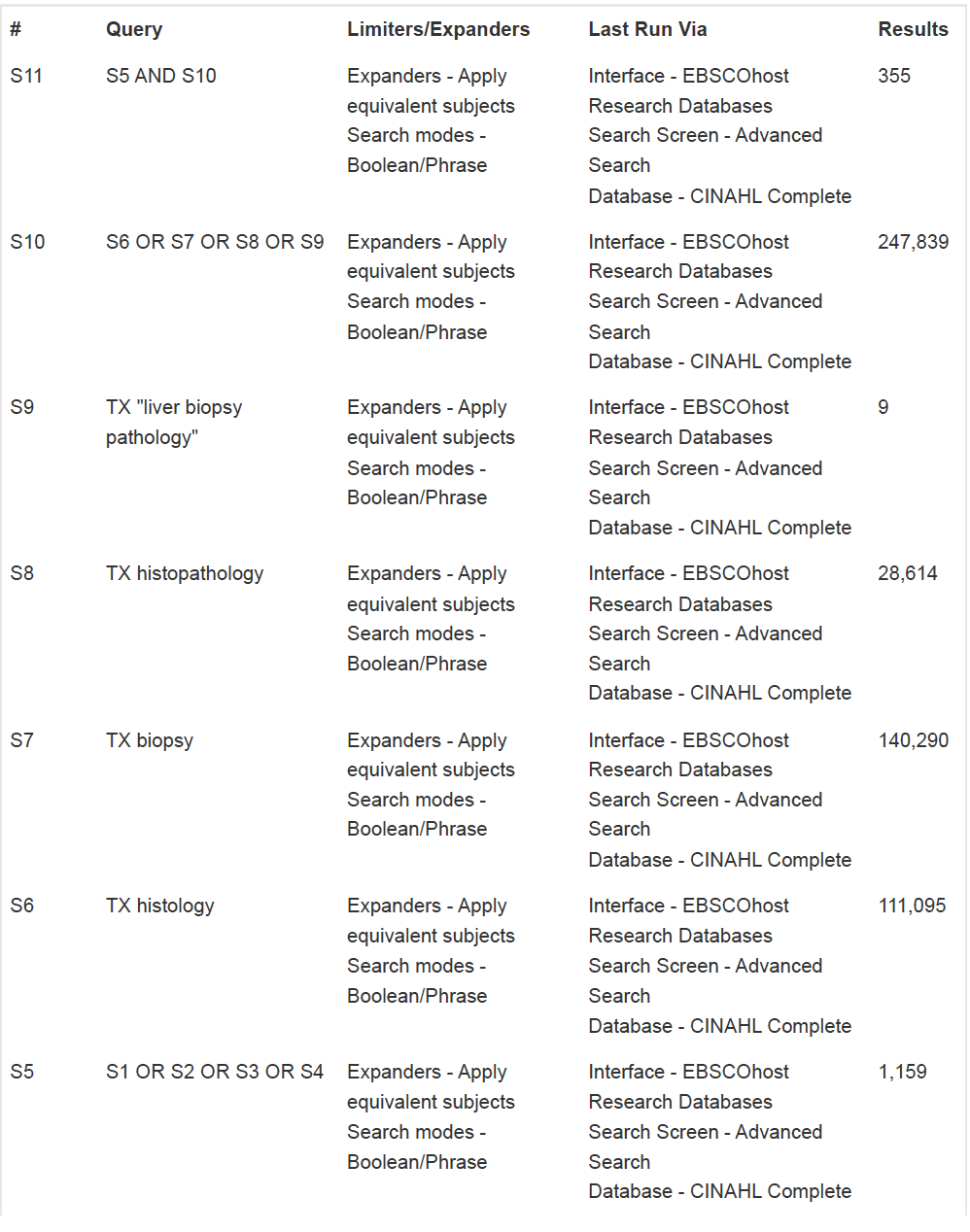

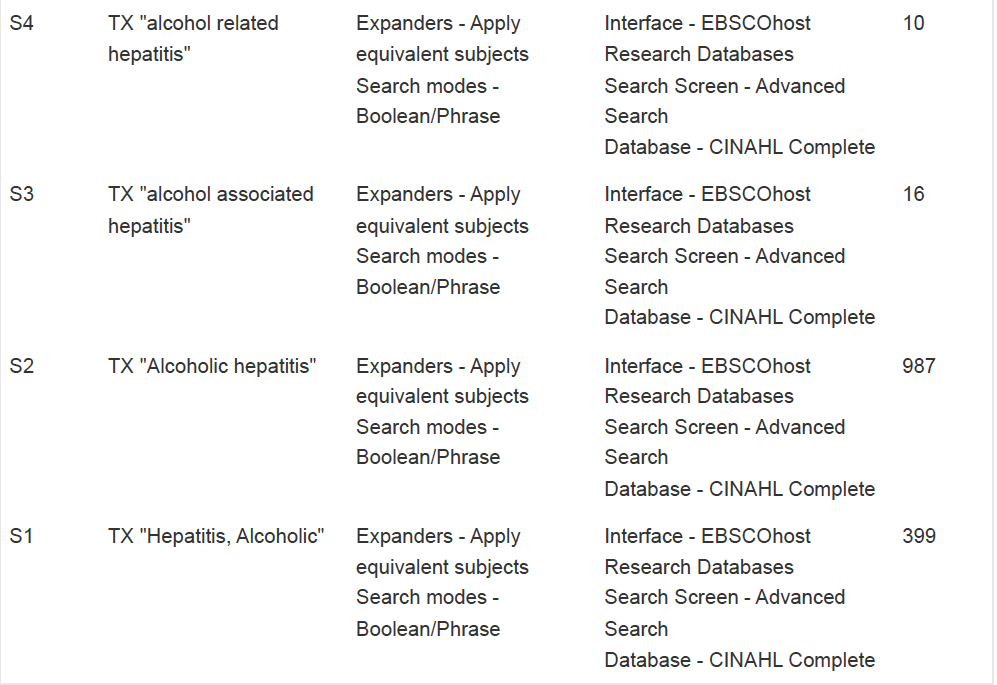


**EMBASE**

**SCOPUS**

**
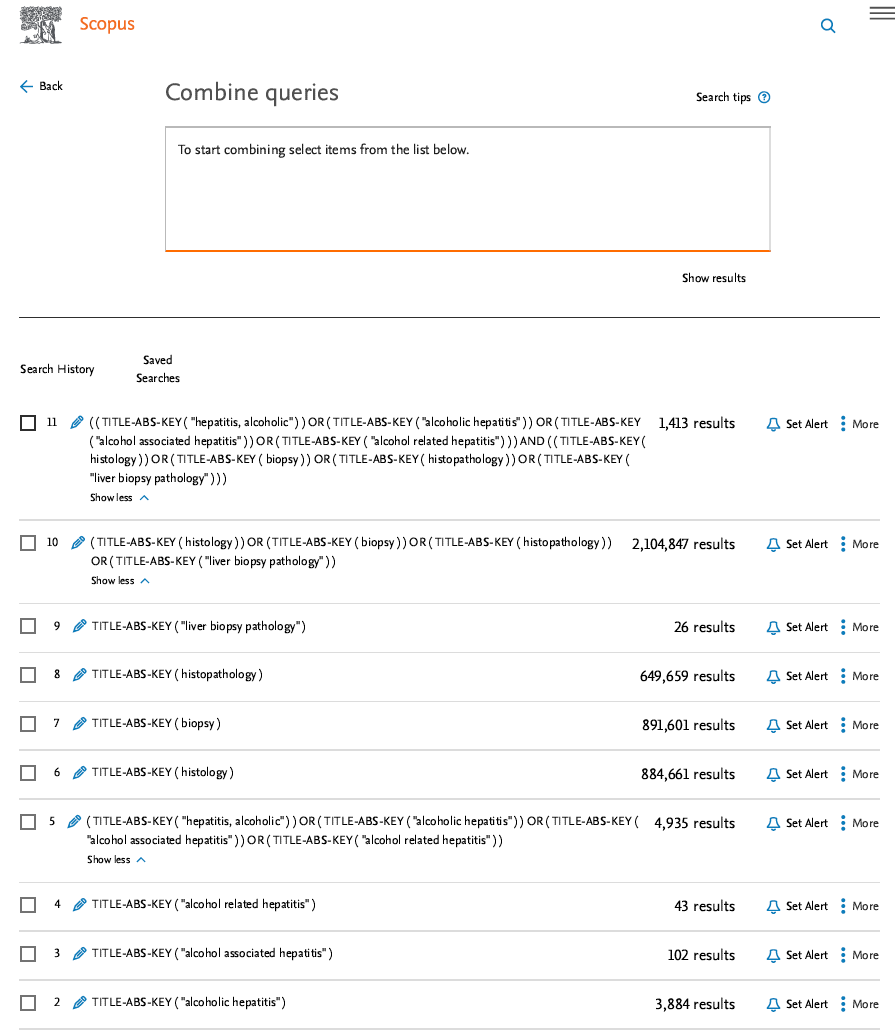
**

**Supplementary Figure 1** Outlier study analysis.

*The x-axis denotes the identity of studies, with corresponding references as follows: 1: Mookerjee 2011, 2: Hardy 2013, 3: Altamirano 2014, 4: Rudler 2015 (PLOS), 5: Rudler 2015 (JHEP), 6: Roth 2017, 7: Shetty 2017, 8: Bissonnette 2017, 9: Shasthry 2018, 10: Choudhary 2018, 11: Lee 2020, 12: Dubois 2020, 13: Atkinson 2020, 14: Forrest 2021, 15: Avitabile 2023.*

*The y-axis represents various influence measures, including:*

- *Studentized residuals (rstudent), where study residuals should ideally follow a normal distribution between two dotted lines.*
- *DFFITS values (dffits), indicating how many standard deviations the predicted average effect for the ith study changes after that study is omitted. Studies are expected to stay above the dotted line.*
- *Cook's distances (cook.d), illustrating how many standard deviations the entire set of predicted values changes when the ith study is left out. Studies should fall below the dotted line.*
- *Covariance ratios (cov.r), where low values suggest studies that, if omitted, would yield more precise parameter estimates.*
- *Leave-one-out estimates of the amount of heterogeneity (tau2.del), showing what τ 2 would be if a study is omitted.*
- *Leave-one-out values of the test statistics for heterogeneity (QE.del), indicating what the χ 2 test for homogeneity would be if a study is omitted.*
- *Hat values (hat), where large values suggest large influence.*
- *Weight, as applicable in the random effects model.*

**Supplementary Figure 2** Leave one out sensitivity analysis depicting the diagnostic precision of clinical criteria of AH.

**Supplementary Figure 3 Funnel plot evaluating the precision of clinical criteria alcohol-associated hepatitis**

*The log-odds of precision and its standard error are presented along the horizontal and vertical axis, respectively, showing asymmetry in the plot (Eggers regression, z = 3.6036, p = 0.0003*


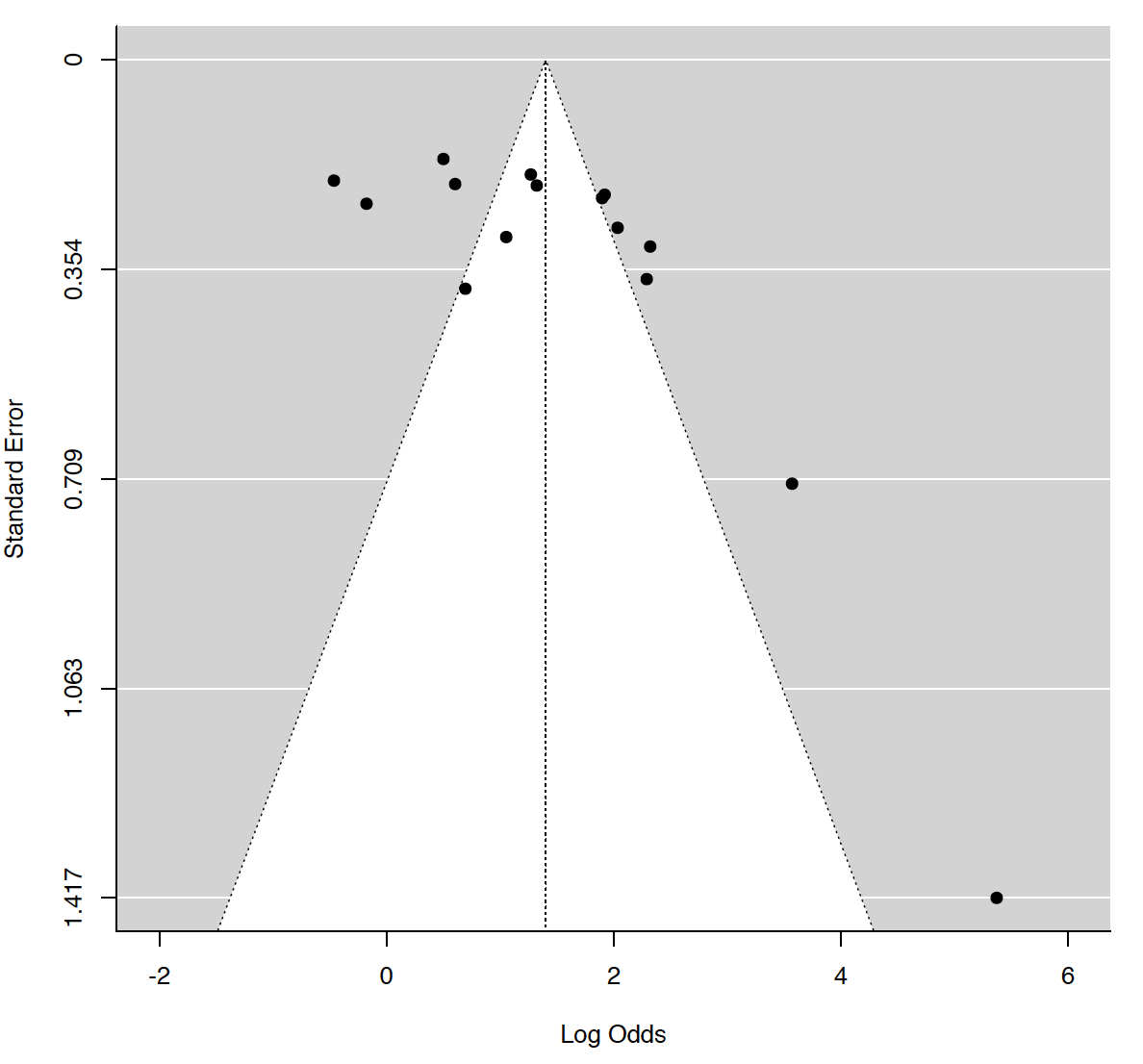


**Supplementary Table 1** Alcoholic hepatitis histologic score (AHHS)^1^

|  |  | **Points** |
| --- | --- | --- |
| **Fibrosis stage** | No/portal fibrosis | 0 |
|  | Expansive fibrosis | 0 |
|  | Bridging fibrosis or cirrhosis | +3 |
| **Bilirubinostasis** | None | 0 |
|  | Hepatocellular only | 0 |
|  | Canalicular or ductular | +1 |
|  | Canalicular or ductular plus hepatocellular | +2 |
| **PMN infiltration** | Mild PMN infiltration | +2 |
|  | Severe PMN infiltration | 0 |
| **Megamitochondria** | No megamitochondria | +2 |
|  | Megamitochondria | 0 |
| AHHS categories (0-9 points) – mild, 0-3; intermediate, 4-5; severe, 6-9 | | |

**Supplementary Table 2** Consortium for the Study of Alcohol-related LiVer disease in Europe (SALVE) Grading

| 1. **Steatosis (S) grade:** Macro vesicular steatosis*; % parenchymal involvement  - Score 0: <5% - Score 1: 5-33% - Score 2: 34-66% - Score 3: >66%  1. **Activity (A) grade:** Sum of scores for hepatocellular and lobular inflammation   *Hepatocellular injury (ballooning (B) or Mallory-Denk bodies (MDB))***   - Score 0: None-rare - Score 1: Few§ - Score 2: Many§§   *Lobular neutrophils (LN)*   - Score 0: None-rare - Score 1: Few§ - Score 2: Many§§ and/or satellitosis%  1. **Cholestasis type**   *Canalicular cholestasis (CC)*   - Score 0: None - Score 1: Present   *Ductular cholestasis (DC)*   - Score 0: None - Score 1: Present |
| --- |
| SALVE grade: S 0-3, A (B/MDB 0-2 + LN 0-2), CC 0-1, DC 0-1  Histological ASH: Ballooning and neutrophil scores >=1 each with activity score>=2 |
| *Adapted from Lackner et al. Journal of Hepatology 2021 vol. 75 j 810–819 *Lipid vacuoles in the cytoplasm of hepatocytes larger than the hepatocellular nucleus. **If scores for ballooning and Mallory-Denk bodies are unequal the higher score is applied. §Feature is appreciated after a reasonable search and is present in few microscopic fields.§§Feature is frequent and easy to find without searching and present in many microscopic fields. %Neutrophils surrounding ballooned hepatocytes.* |

| **Supplementary table 3: Consortium for the Study of Alcohol-related LiVer disease in Europe (SALVE) staging** | | |
| --- | --- | --- |
| **SFS** | **Description** | **Criteria** |
| 0 | No fibrosis | Fibrosis is absent |
| 1 | Mild fibrosis | Periportal fibrosis only or PCF^b^ in zone(s) 3 ± 2 |
|  |  | SFS 1P^c^: PCF in zone(s) 3 ± 2 only |
| 2 | Moderate fibrosis | Periportal fibrosis and PCF in zone(s)  3 ± 2 |
| 3 | Severe fibrosis | >−1 complete septum^d^ bridging portal tracts and/or central veins, ±PCF  SFS 3P^c^: Panlobular PCF and/or complete septal PCF ± few dense septa ± venous lesions |
| 4A | Cirrhosis thin septa | >−1 parenchymal nodule^e^, thin septa^f^,  ± 1 broad septum^g^, ±PCF  SFS 4AP^c^: Severe PCF^h^ in >50% of parenchyma, indistinct parenchymal nodules^i^ |
| 4B | Cirrhosis broad septa | Parenchymal nodules, >1 broad septum, ± 1 very broad septum^j^, ±PCF  SFS 4BP^c^: Severe PCF in >50% of parenchyma |
| 4C | Cirrhosis very broad septa | Parenchymal nodules, >1 very broad septum, ±PCF SFS 4CP^c^: Severe PCF in >50% of parenchyma |
| *Adapted from Lackner et al. Journal of Hepatology 2021 vol. 75 j 810–819*  *PCF, pericellular fibrosis; SALVE, Consortium for the Study of Alcohol-related LiVer disease in Europe; SFS, SALVE Fibrosis Stage. ^a^Description of the full range of topographical abnormal fibrosis including the degree of both dense septal and pericellular fibrosis. ^b^Pericellular fibrosis: Collagen fibers surrounding single or small groups of hepatocytes. ^c^OPTIONAL, the presence of pericellular fibrosis as a dominant fibrosis type may be classified as “P” substage.*  *^d^Complete septum: Fibrous band consisting mainly of collagen fibers resembling septa in viral hepatitis or septal PCF crossing biopsy diameter and linking portal tracts, portal tracts and central veins, or central veins.*  *eParenchymal nodule without evidence of portal-central relations surrounded by dense septa.*  *fThin septum: Dense septum, <50% of diameter of smallest parenchymal nodule.*  *^g^Broad septum: Dense septum, >−50% of the diameter of smallest parenchymal nodule but not thicker.*  *^h^PCF evaluated at LOW magnification (20x or 40x total magnification).*  *iParenchymal areas of indistinct nodular shape dissected by severe PCF.*  *jVery broad septum: Dense septum, wider than the diameter of smallest parenchymal nodule.* | | |

**Supplementary Table 4** Additional characteristics of included studies

| **Study** | **Cohort characteristics** | **N** | **Age (SD)** | **Male (%)** | **MDF (SD)** | **MELD (SD)** | **CTP** | **Bil (SD)** | **AST (SD)** | **ALT (SD)** | **WBC** | **INR** | **Creat** | **Alb** | **Ascites (%)** | **Bleed (%)** | **HE (%)** |  |
| --- | --- | --- | --- | --- | --- | --- | --- | --- | --- | --- | --- | --- | --- | --- | --- | --- | --- | --- |
| Mookerjee 2011^2^ | Clinical AH | 68 | 51.0 (26.1) | 52.0 | 38.0 (30.3) | 12.5 (11.4) | 11.2 | 13.3 (10.6) |  |  | 13.3 | 1.7 | 1.1 | 2.5 | 24.0 |  | 27.0 |  |
| Hardy 2013^3^ | Clinical AH | 58 | 46.1 (6.6) | 59.5 | 75.5 (NA) |  | 11.7 | 21.5 (NA) |  | 38.9 (NA) | 11.6 | 2.1 | 1.6 | 2.8 |  |  |  |  |
| Altamirano 2014^1^ | Biopsy AH | 121 | 47.9 (9.8) | 78.0 |  | 17.3 (7.5) | 11.4 | 10.6 (10.0) | 132.0 (79.5) | 52.4 (27.0) | 8.9 | 1.6 | 0.8 | 2.7 | 82.0 | 26.0 | 17.0 |  |
| Rudler 2015 plos^4^ | Clinical AH | 123 | 56.7 (8.8) | 75.0 | 62.7 (31.4) | 20.7 (7.0) |  |  |  |  |  |  |  |  |  | 50.0 |  |  |
| Rudler 2015 jhep^5^ | Biopsy AH | 105 | 54.6 (9.1) | 76.5 | 65.1 (31.2) | 25.0 (5.3) | 11.4 | 10.5 (7.8) |  |  | 9.9 | 2.4 | 1.0 | 2.6 | 70.5 | 29.3 | 46.1 |  |
| Roth 2017^6^ | Clinical AH | 172 | 44.5 (9.1) | 65.5 | 52.4 (19.3) | 18.4 (3.7) |  | 21.1 (9.4) | 174.7 (84.5) | 57.2 (33.9) | 11.4 | 1.8 | 0.7 | 2.8 | 64.0 |  |  |  |
| Shetty 2017^7^ | Clinical AH | 30 | 45.3 (9.8) |  |  |  |  | 22.9 (NA) |  |  | 16.0 | 2.2 | 1.0 | 2.8 | 7.0 |  |  |  |
| Bissonnette 2017^8^ | Clinical AH | 151 | 53.4 (8.1) | 79.0 | 49.4 (31.4) | 18.9 (7.1) |  | 7.1 (7.6) | 106.5 (69.6) |  | 8.3 | 1.7 | 0.8 | 2.5 |  |  |  |  |
| Shasthry 2018^9^ | Biopsy AH | 71 | 39.9 (10.0) |  | 79.0 (26.5) | 25.1 (4.0) | 11.1 | 20.3 (9.5) |  |  | 15.7 | 2.1 | 0.5 | 2.3 |  |  |  |  |
| Choudhary 2018^10^ | Biopsy AH | 39 | 43.2 (8.5) |  | 54.8 (26.9) | 22.1 (4.5) | 10.9 | 10.5 (6.3) |  |  |  | 2.0 |  | 2.9 | 100.0 | 18.0 | 61.0 |  |
| Lee 2020^11^ | Biopsy AH | 107 | 51.3 (12.0) | 79.4 | 17.4 (27.1) | 14.7 (10.5) |  | 4.6 (5.6) | 95.9 (63.1) | 37.3 (26.3) | 6.1 | 1.3 | 0.7 | 3.3 | 43.0 |  | 1.0 |  |
| Dubois 2020^12^ | Clinical AH | 107 | 54.0 (3.0) | 62.0 | 61.6 (8.3) | 22.6 (2.3) | 10.0 | 13.7 (3.8) |  |  |  | 1.7 | 0.7 | 2.7 |  |  |  |  |
| Atkinson 2020^13^ | Clinical AH | 87 | 47.8 (8.5) | 69.0 | 58.1 (27.1) | 24.3 (5.2) |  | 18.7 (11.4) | 119.3 (51.3) | 41.3 (18.0) | 9.5 | 1.7 | 0.8 | 2.6 |  |  | 27.0 |  |
| Forrest 2021^14^ | Clinical AH | 161 |  |  |  |  |  | 19.9 (2.2) |  |  |  | 1.8 | 0.8 |  |  |  |  |  |
| Avitabile 2023^15^ | Clinical AH | 168 | 54.7 (10.9) | 70.5 | 30.4 (25.1) | 16.0 (7.1) | 8.5 | 5.1 (5.1) |  |  | 6.9 | 1.4 | 0.8 | 3.1 | 56.0 | 9.5 | 23.0 |  |
| *AH: alcohol-associated hepatitis, SD: standard deviation, MDF: Maddrey’s discriminant function, MELD: Model for End stage Liver Disease, CTP: Child-Turcotte-Pugh score, Bil: total bilirubin, AST: Aspartate aminotransferase, ALT: Alanine aminotransferase, WBC: white blood cell count, INR: International normalised ratio, Creat: creatinine, Alb: albumin, HE: hepatic encephalopathy* | | | | | | | | | | | | | | | | | | |

**Supplementary Table 5** Biopsy findings in the cohort NOT identified as alcoholic hepatitis on liver histology.

| **Study** | **Cohort** | **Biopsy findings** | **Percentage** |
| --- | --- | --- | --- |
| Bissonnette 2017^8^ | Test cohort (n=37) | Alcoholic cirrhosis | 95 |
|  |  | Steatosis with advanced fibrosis | 2.7 |
|  |  | Cholestasis with features suggestive of biliary salt transporter gene mutation | 2.7 |
|  | Validation cohort (n=20) | Alcoholic cirrhosis | 70 |
|  |  | Hypoxic hepatitis | 14 |
|  |  | Alcoholic foamy degeneration | 10 |
|  |  | Acute infectious mononucleosis hepatitis | 5 |
|  |  |  |  |
| Roth 2017^6^ | Derivation cohort (n=10) | Alcoholic foamy degeneration | 60 |
|  |  | Alcoholic fatty liver with jaundice | 40 |
|  |  |  |  |
| Shetty 2017^7^ | n=10 | Alcoholic cirrhosis | 100 |
|  |  |  |  |
| Hardy 2013^3^ | n=15 | Alcoholic cirrhosis | 100 |
|  |  |  |  |
| Forrest 2021^14^ | n=21 | Alcohol related liver injury (without ballooning) | 85.71 |
|  |  | No alcohol related liver injury | 14.28 |

**Supplementary Table 6** Risk of bias of the included studies using the QUADAS-2 tool.

| **ID** | **Domain 1: Patient Selection** | | | | | **Domain 2: Index Test** | | | **Domain 3: Reference Standard** | | | **Domain 4: Patient Flow** | **Overall** |
| --- | --- | --- | --- | --- | --- | --- | --- | --- | --- | --- | --- | --- | --- |
| **Study** | **Random Sample Enrolled** | **Case-Control Avoided** | **Avoid Inappropriate Exclusions** | **ROB** | **Applicability Concern** | **Criteria** | **ROB** | **Applicability Concern** | **Criteria** | **ROB** | **Applicability Concern** | **ROB** | **ROB** |
| Mookerjee 2011^2^ | Yes | Yes | No | Low | Low | EASL 2012 | High | Low | Two Blinded Pathologists | Low | Low | Low | Low |
| Hardy 2013^3^ | Yes | No | No | High (select cases with DF>32) | Low | EASL 2012 | High | Low | Single expert pathologist | Low | Low | Unclear | High |
| Altamirano 2014^1^ | Yes | Yes | Yes | Low | Low | EASL 2012 | High | Low | Single blinded liver pathologist | Low | Low | Low | Low |
| Rudler 2015 Plos^4^ | Yes | Yes | No | High (select cases with DF>32) | Low | EASL 2012 | High | Low | Single blinded pathologist | Low | Low | Low | High |
| Rudler 2015 Jhep^5^ | No | No | No | High (select cases with DF>32) | Low | EASL 2012 | Low | Low | Unclear | Unclear | Unclear | High | High |
| Roth 2017^6^ | No | Yes | No | High (select cases with DF>32) | Low | Classical criteria | Low | Low | Single expert blinded liver pathologist In Derivation Cohort, Two expert liver pathologists In Validation Cohort | Low | Low | Low | Low |
| Shetty 2017^7^ | No | Yes | No | High (select cases with DF>32) | Low | NIAAA | Low | Low | Single Pathologist | High | High | High | High |
| Bissonnette 2017^8^ | Yes | Yes | Yes | Low | Low | EASL 2012 | High | Low | Three expert liver pathologists | Low | Low | Low | Low |
| Shasthry 2018^9^ | No | Yes | No | High (select population) | Low | Classical criteria | Low | Low | Two blinded liver pathologists | Low | Low | Low | Low |
| Choudhary 2018^10^ | No | Yes | Yes | High (retrospective, select population) | High | NIAAA | Low | Low | No Details About Pathologist | Unclear | Unclear | High | High |
| Lee 2020^11^ | Yes | Yes | Yes | Low | Low | Unclear | Unclear | Unclear | Single expert liver pathologist | Low | Low | Low | Low |
| Dubois 2020^12^ | Yes | Yes | No | Low | Low | NIAAA | Low | Low | Two expert liver pathologists | Low | Low | Low | Low |
| Atkinson 2020^13^ | No | No | No | High (select cases with DF>32) | Low | Classical criteria | Low | Low | Two expert blinded liver pathologists | Low | Low | Unclear | Low |
| Forrest 2021^14^ | No | Yes | No | High (select cases with DF>32) | Low | Classical criteria | Low | Low | Two expert blinded liver pathologists | Low | Low | Low | Low |
| Avitabile 2023^15^ | Yes | Yes | Yes | Low | Low | New NIAAA | Low | Low | Two expert liver pathologists | Low | Low | Low | Low |
| ROB: Risk of bias as per QUADAS2 tool | | | | | | | | | | | | | |

**REFERENCES**

1. Altamirano J, Miquel R, Katoonizadeh A, et al. A Histologic Scoring System for Prognosis of Patients With Alcoholic Hepatitis. *Gastroenterology*. 2014;146(5):1231-1239.e6. doi:10.1053/j.gastro.2014.01.018

2. Mookerjee RP, Lackner C, Stauber R, et al. The role of liver biopsy in the diagnosis and prognosis of patients with acute deterioration of alcoholic cirrhosis. *J Hepatol*. 2011;55(5):1103-1111. doi:10.1016/j.jhep.2011.02.021

3. Hardy T, Wells C, Kendrick S, et al. White cell count and platelet count associate with histological alcoholic hepatitis in jaundiced harmful drinkers. *BMC Gastroenterol*. 2013;13(1):55. doi:10.1186/1471-230X-13-55

4. Rudler M, Mouri S, Charlotte F, et al. Validation of AshTest as a Non-Invasive Alternative to Transjugular Liver Biopsy in Patients with Suspected Severe Acute Alcoholic Hepatitis. Sookoian SC, ed. *PLOS ONE*. 2015;10(8):e0134302. doi:10.1371/journal.pone.0134302

5. Rudler M, Mouri S, Charlotte F, et al. Prognosis of treated severe alcoholic hepatitis in patients with gastrointestinal bleeding. *J Hepatol*. 2015;62(4):816-821. doi:10.1016/j.jhep.2014.11.003

6. Roth NC, Saberi B, Macklin J, et al. Prediction of histologic alcoholic hepatitis based on clinical presentation limits the need for liver biopsy. *Hepatol Commun*. 2017;1(10):1070-1084. doi:10.1002/hep4.1119

7. Shetty S, Venkatakrishnan L, Krishanveni J, Kumari S. Transjugular liver biopsy in severe alcoholic hepatitis. *Indian J Gastroenterol*. 2017;36(1):23-26. doi:10.1007/s12664-016-0720-6

8. Bissonnette J, Altamirano J, Devue C, et al. A prospective study of the utility of plasma biomarkers to diagnose alcoholic hepatitis. *Hepatology*. 2017;66(2):555-563. doi:10.1002/hep.29080

9. Shasthry SM, Rastogi A, Bihari C, et al. Histological activity score on baseline liver biopsy can predict non-response to steroids in patients with severe alcoholic hepatitis. *Virchows Arch*. 2018;472(4):667-675. doi:10.1007/s00428-018-2330-4

10. Choudhary NS, Saigal S, Gautam D, et al. Good outcome of living donor liver transplantation for severe alcoholic hepatitis not responding to medical management: A single center experience of 39 patients. *Alcohol*. 2019;77:27-30. doi:10.1016/j.alcohol.2018.07.009

11. Lee DH, Choi YI, Bae JM, et al. Prognostic Value of the Alcoholic Hepatitis Histologic Score in Korean Patients with Biopsy-Proven Alcoholic Hepatitis. *Gut Liver*. 2020;14(5):636-643. doi:10.5009/gnl19203

12. Dubois M, Sciarra A, Trépo E, et al. Histologic parameter score does not predict short‐term survival in severe alcoholic hepatitis. *United Eur Gastroenterol J*. 2020;8(9):1003-1012. doi:10.1177/2050640620949737

13. Atkinson SR, Grove JI, Liebig S, et al. In Severe Alcoholic Hepatitis, Serum Keratin-18 Fragments Are Diagnostic, Prognostic, and Theragnostic Biomarkers. *Am J Gastroenterol*. 2020;115(11):1857-1868. doi:10.14309/ajg.0000000000000912

14. Forrest E, Petts G, Austin A, et al. The diagnostic and prognostic significance of liver histology in alcoholic hepatitis. *Aliment Pharmacol Ther*. 2021;53(3):426-431. doi:10.1111/apt.16157

15. Avitabile E, Díaz A, Montironi C, et al. Adding Inflammatory Markers and Refining National Institute on Alcohol Abuse and Alcoholism Criteria Improve Diagnostic Accuracy for Alcohol-associated Hepatitis. *Clin Gastroenterol Hepatol*. Published online March 2023:S1542356523002318. doi:10.1016/j.cgh.2023.03.023

16. Andrade P, Silva M, Rodrigues S, Lopes J, Lopes S, Macedo G. Alcoholic hepatitis histological score has high accuracy to predict 90-day mortality and response to steroids. *Dig Liver Dis*. 2016;48(6):656-660. doi:10.1016/j.dld.2016.03.002

17. Lackner C, Stauber RE, Davies S, et al. Development and prognostic relevance of a histologic grading and staging system for alcohol-related liver disease. *J Hepatol*. 2021;75(4):810-819. doi:10.1016/j.jhep.2021.05.029
